# QRStree: A prefix tree-based model to fetal QRS complexes detection

**DOI:** 10.1371/journal.pone.0223057

**Published:** 2019-10-01

**Authors:** Wei Zhong, Xuemei Guo, Guoli Wang

**Affiliations:** 1 School of Data and Computer Science, Sun Yat-sen University, Guangzhou, China; 2 Key Laboratory of Machine Intelligence and Advanced Computing, Ministry of Education, Guangzhou, China; Aristotle University of Thessaloniki, GREECE

## Abstract

Non-invasive fetal electrocardiography (NI-FECG) plays an important role in fetal heart rate (FHR) measurement during the pregnancy. However, despite the large number of methods that have been proposed for adult ECG signal processing, the analysis of NI-FECG remains challenging and largely unexplored. In this study, we propose a prefix tree-based framework, called QRStree, for FHR measurement directly from the abdominal ECG (AECG). The procedure is composed of three stages: Firstly, a preprocessing stage is employed for noise elimination. Secondly, the proposed prefix tree-based method is used for fetal QRS complexes (FQRS) detection. Finally, a correction stage is applied for false positive and false negative correction. The novelty of the framework relies on using the range of FHR to establish the connections between the FQRS. The consecutive FQRS can be considered as strings composed of alphabet items, thus we can use the prefix tree to store them. A vertex of the tree contains an alphabet, thus a path of the tree gives a string. Such that, by storing the connections of the FQRS into the prefix tree structure, the problem of FQRS detection converts to a problem of optimal path selection. Specifically, after selecting the optimal path of the tree, the nodes in the optimal path are collected as detected FQRS. Since the prefix tree can cover every possible combination of the FQRS candidates, it has the potential to reduce the occurrence of miss detections. Results on two different databases show that the proposed method is effective in FHR measurement from single-channel AECG. The focus on single-channel FHR measurement facilitates the long-term monitoring for healthcare at home.

## Introduction

**Fig 1:**
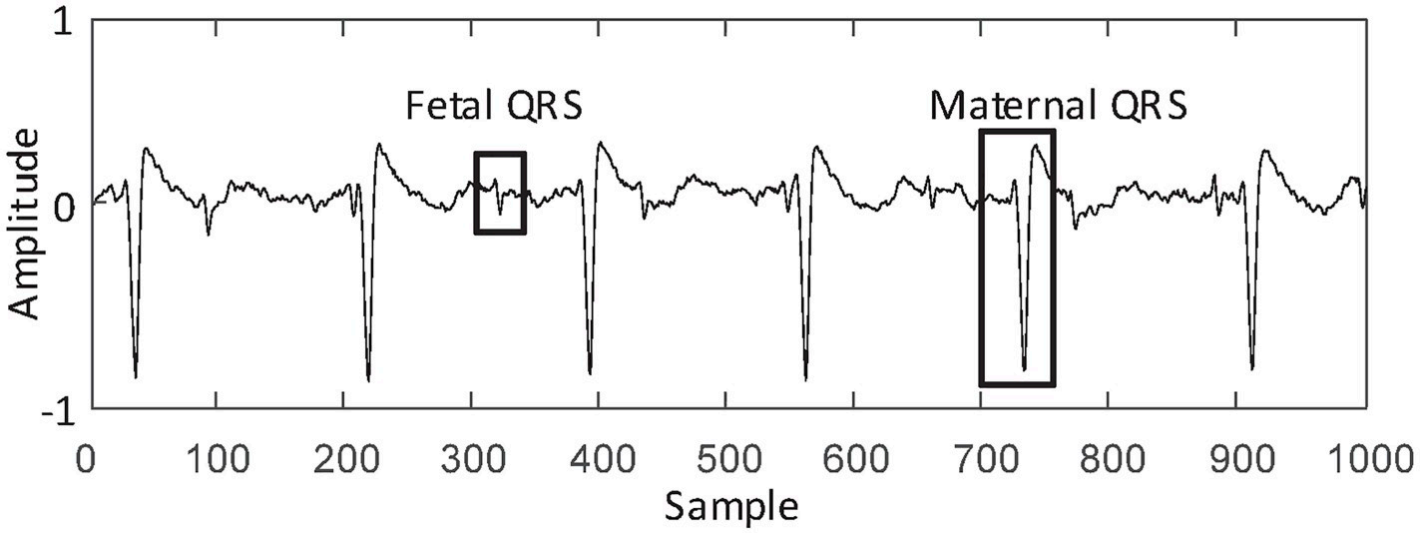
Illustration of AECG. On the AECG, the maternal QRS and fetal QRS are marked with rectangles. It is noted that the MECG has a much larger amplitude than the FECG.

Non-invasive fetal electrocardiography (NI-FECG) can be used for fetal heart rate (FHR) measurement throughout the pregnancy [1–4]. However, extracting the FHR information from the abdominal ECG (AECG) remains a challenging task [5–7]. On the one hand, the AECG collected from the abdomen is inevitably affected by a variety of noise interferences [8]. On the other hand, the maternal ECG (MECG) component of the AECG is the predominant interference source, which has a much larger amplitude than the fetal ECG (FECG) (see Fig 1) [9–11]. This paper addresses the issue of detecting the location of fetal QRS complexes (FQRS) for FHR measurement.

In order to obtain a reliable FHR measurement, the location of FQRS is the primary feature that any approach must achieve from the AECG [1, 12, 13]. However, despite the significant advances in the field of adult QRS detection, the analysis of FQRS detection remains largely unexplored [14–16]. Unlike the adult QRS which can be directly detected from the AECG, the FQRS is usually detected after a procedure of MECG elimination [17, 18]. For this purpose, a considerable amount of literature has been published to remove the MECG from the AECG for FHR monitoring [1, 19], such as the blind source separation (BSS) methods [17, 20, 21], the adaptive noise cancelling (ANC) methods [22–24], and the template subtraction (TS) methods [25–27]. Although the techniques based on the separation or cancellation of MECG make FHR measurement possible, the FHR outcome highly depends on the performance of MECG elimination, that is, a reliable FHR is hard to be obtained when the MECG is not completely removed, what is more, the FECG signal is significantly distorted after suppressing the MECG [23, 28]. As discussed in [29], the morphology of cardiac electrical signals contains a lot of information related to cardiac defects. Recently, the work of [30] presents a segmentation-based method to detect the FQRS from single-channel AECG. It uses a convolutional neural network to distinguish whether the segmented AECG contains FQRS. In this work, after analyzing graphical representation of the FQRS, we propose a prefix tree-based framework, called QRStree, for FHR measurement without separation of FECG.

**Fig 2:**
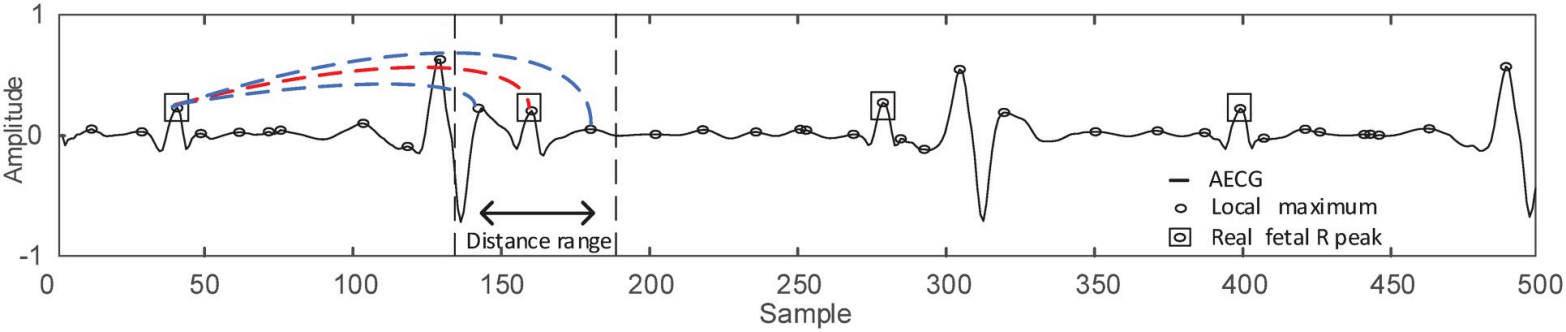
Illustration of the connections between two adjacent FQRS on the AECG. Given the range of FHR, the distance range of the next FQRS can be determined. For the first FQRS, three local maxima are within the distance range, such that these local maxima are connected with the first FQRS (one red line and two blue lines). The red line indicates the connection between two real FQRS.

As shown in Fig 2, the heart beats of the fetus are inherently sequential, and the fetal R peaks exist in the local maxima. We can use the range of the FHR to obtain the distance range between the fetal R peaks (defined in Eq (2)). Such that the consecutive FQRS can be connected by the distance range. For the purpose of illustration, an example of connected FQRS is shown in Fig 2. Here, we use a prefix tree structure to store these sequential connections. The prefix tree, also called trie, is a useful data structure to store dynamic sets such as strings and sequences [31–33]. It is widely used in the field of analyzing data characteristics or gaining information needed for decision-making [34–36]. The consecutive FQRS can be considered as strings composed of alphabet items, thus we can use the prefix tree to store them. A vertex of the tree contains an alphabet, thus a path of the tree gives a string. Each string is represented as the path from a representative vertex to the root. Such that, after storing the sequential connections into the prefix tree structure, the problem of FQRS detection converts to a problem of optimal path selection. Specifically, by analyzing the graphical representation of the paths, the optimal path of the tree can be selected, and the nodes in the optimal path are collected as detected FQRS.

In this work, the proposed method is compared with five single-channel methods. These methods include four TS methods [25–27, 37] and one segmentation-based method [30]. The experimental results show that the proposed method is effective in FQRS detection. The prefix tree-based framework has the following advantages:

- Firstly, since the prefix tree can cover every possible combination of the FQRS candidates, it has the potential to reduce the occurrence of miss detections.
- Secondly, since the tree structure is built on the range of FHR, it will not be affected by the large amplitude of MECG.
- Finally, although the FECG and MECG overlap in both time and frequency domains [23], only the local maxima satisfied the range of FHR are collected to construct the tree. And the model can skip the maternal R peaks which dissatisfy the range of FHR. Such that the FQRS and MQRS are ‘heart rate separable’.

The details of the proposed method are described in the following sections.

## Materials and methods

### Basic structure

**Fig 3:**
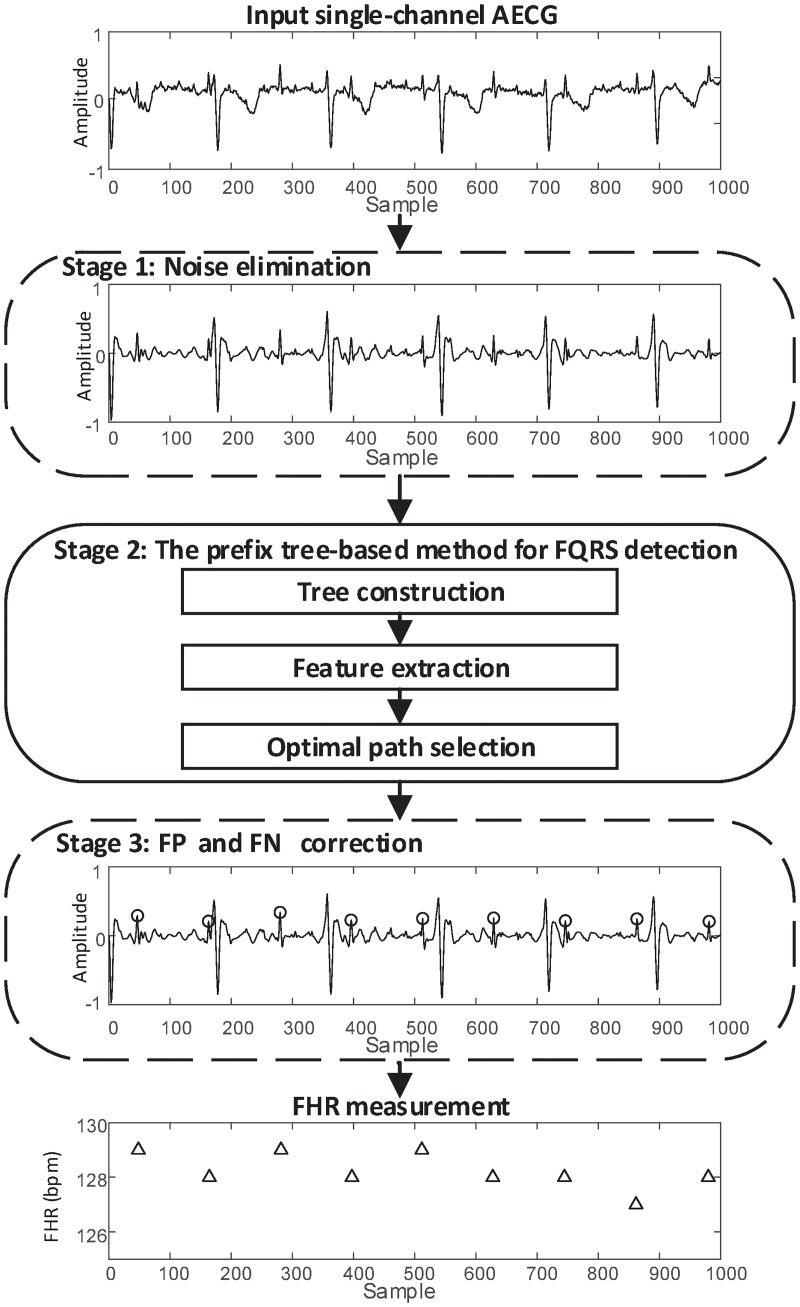
The procedure of the proposed method for FHR monitoring. Among the three stages, the prefix tree is constructed in stage 2.

Unlike the TS methods which are based on the elimination of MECG, we propose a novel method to detect the location of FQRS directly from the AECG. As shown in Fig 3, the procedure of the proposed method mainly consists of three stages. Stage 1: Noise elimination; Stage 2: The prefix tree-based method for FQRS detection; Stage 3: False positive (FP) and false negative (FN) correction. The details of each stage are described in the following subsections.

### Stage 1: Noise elimination

The AECG is inevitably affected by noise. In order to remove the noise, a preprocessing stage is widely used in the previous approaches such as the TS methods. In stage 1, inspired by the work of [30], a band-pass Butterworth filter (between 0.5 Hz and 100 Hz) is applied to remove the baseline wander and the high-frequency noise above 100 Hz.

### Stage 2: The prefix tree-based method for FQRS detection

After the stage of noise elimination, a new method based on the prefix tree structure is implemented to carry out the FQRS detection. This method contains three steps. In step 1, considering the range of the FHR, the tree contained the location of candidate FQRS is structured. In step 2, the features of the nodes (single FQRS candidate) and the features of the paths (consecutive FQRS candidates) are extracted. In step 3, the optimal path of the tree is selected, then the location of the FQRS can be detected. In this study, each FQRS candidate can be considered as an alphabet, thus the consecutive FQRS is considered as strings composed of alphabet items. The prefix tree is a tree shaped data structure widely used in storing strings. Here, three characteristics of the prefix tree are listed:

- A vertex of the tree has an alphabet, thus a path of the tree gives a string.
- Each string is represented as the path from a representative vertex to the root.
- Every string in the tree is unique.

These characteristics are used in the construction of the prefix tree.

#### Step 1: Tree construction

**Fig 4:**
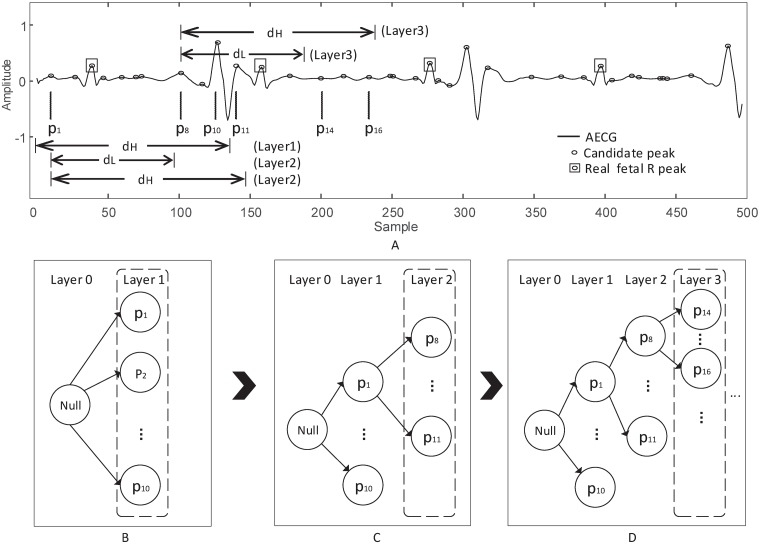
The procedure of tree construction. The prefix tree is constructed layer by layer.

Fig 4 shows the procedure of tree construction. As shown in Fig 4A, the real fetal R peaks are scattered in the local maxima. The goal of the proposed method is to find out the real fetal R peaks from the candidate peaks. In this step, the prefix tree based on the limit of the timed distance between the FQRS is structured.

The timed distance of two detected R peaks *RR* is defined as

$$RR_{i}=R_{i+1}-R_{i}$$ (1)

where *R*_*i*_ is the timed position of the *i*-th detected R peak.

Given the range of FHR *f* ∈ (*f*_*L*_, *f*_*H*_) *bpm*, the range of the distance between two fetal R peaks *d* ∈ (*d*_*L*_, *d*_*H*_) *s* could be defined by

d=60/f*fs (2)

where *f* is the FHR, where *fs* is the sampling frequency.

The prefix tree is constructed layer by layer. As shown in Fig 4B, the prefix tree starts with the root at layer 0 and with a null value. In order to construct the layer 1 of the tree (see Fig 4A and 4B), the local maxima at the beginning of AECG are evaluated by

$$p_{j}<d_{H}$$ (3)

where *p*_*j*_ is the timed position of the *j*-th local maximum. Then the local maxima satisfied the Eq (3) are collected to use as the nodes in the layer 1. As shown in Fig 4B, the *p*_1_, *p*_2_, …, *p*_10_ are used as the nodes in the layer 1. Every node contains one FQRS candidate.

In order to construct the layer *k* (*k* ≥ 2) of the tree (see Fig 4A, 4C and 4D), the limit of RR distance based on the FHR is employed. Specifically, the local maxima are evaluated by

$$d_{L}<RR_{k-1}<d_{H}$$ (4)

then the local maxima satisfied the Eq (4) can be saved as the fetal R peak candidates for layer *k* (*k* ≥ 2). As shown in Fig 4C, the *p*_8_, *p*_9_, …, *p*_11_ are used as the nodes in the layer 2 of *p*_1_. After the node *p*_1_, the *p*_14_, *p*_15_, *p*_16_ are used as the nodes in the layer 3 of *p*_8_ (see Fig 4D). By using these strategies, a multilayer tree could be structured.

#### Step 2: Feature extraction

In this step, the features of the nodes (single FQRS candidate) and the features of the paths (consecutive FQRS candidates) are extracted. In a *β*-layer tree (structured in step 1 of stage 2), each path includes *β* fetal R peak candidates. As shown in Fig 4D, besides the root, the *path*_1,8,14_ contains three fetal R peak candidates (*p*_1_, *p*_8_, *p*_14_).

**Fig 5:**
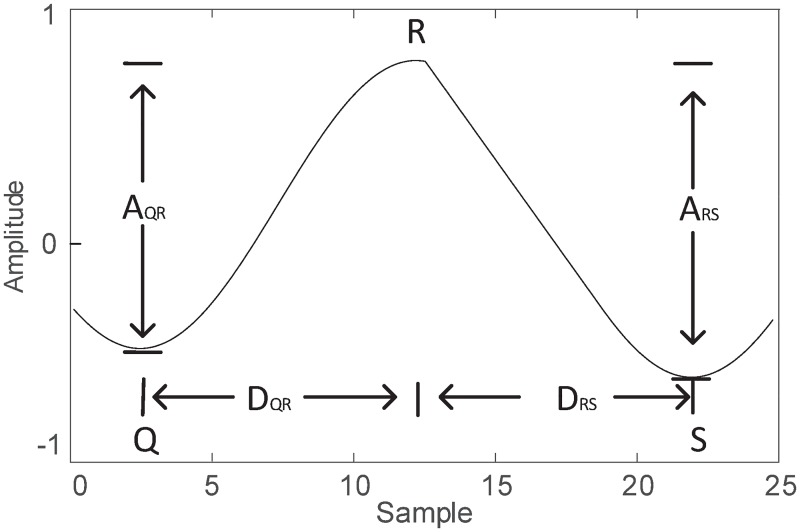
The feature representation of FQRS. Each FQRS is composed of a local minimum (Q-peak candidate), a local maximum (R-peak candidate) and a local minimum (S-peak candidate).

A fetal R peak candidate is composed of a local minimum (Q-peak candidate), a local maximum (R-peak candidate) and a local minimum (S-peak candidate). As shown in Fig 5, three features of the single FQRS candidate are collected, namely the ratio of RS amplitude to RS distance *R*_*RS*_ (*R*_*RS*_ = *A*_*RS*_/*D*_*RS*_), amplitude of RS (*A*_*RS*_), the ratio of QR amplitude to QR distance *R*_*QR*_ (*R*_*QR*_ = *A*_*QR*_/*D*_*QR*_).

By analyzing the path-wise representation of FQRS candidates in the tree, three regular patterns of the inter-QRS correlation can be noted.

- In this work, we use the variances of *R*_*QR*_ and *R*_*RS*_ to represent the graphical similarities between the local maxima in a path. It is noted that the graphical similarities between the fetal R peaks are higher than the noise.
- In some cases, the values of single noise peak are larger than the real fetal R peak in terms of *A*_*RS*_, *R*_*QR*_ and *R*_*RS*_. However, the overall values (e.g., median) of the real fetal R peaks in a path are larger than the noise in these terms.
- The RR distances of the real fetal R peaks are relatively stable in a short time.

Therefore, six features of the paths (consecutive FQRS candidates) are retained to represent the regular patterns.

- *F*_*QR*_: the variance of *R*_*QR*_.
- *F*_*RS*_: the variance of *R*_*RS*_.
- *F*_*Am*_: the median of *A*_*RS*_.
- *F*_*QRm*_: the median of *R*_*QR*_.
- *F*_*RSm*_: the median of *R*_*RS*_.
- *F*_*RR*_: the variance of RR distances.

The variance is given by

$$v^{2}=\frac{\sum_{n=1}^{N}{\left(x_{i}-\mu \right)}^{2}}{N}$$ (5)

where N corresponds to the number of fetal R peak candidates in a path, where *μ* corresponds to the mean value of the feature. The *F*_*QR*_ and *F*_*RS*_ represent the graphical similarity between the fetal R peak candidates in a path. The lower variance value shows a higher graphical similarity. And the greater median value shows a greater probability of being a path of real fetal R peaks. In addition, the *F*_*RR*_ represents the stability of the RR distances in a path.

#### Step 3: Optimal path selection

After extracting the six features of paths (*F*_*QR*_, *F*_*RS*_, *F*_*Am*_, *F*_*QRm*_, *F*_*RSm*_ and *F*_*RR*_), effective techniques are needed to select the optimal path for fetal QRS detection.

In this step, a robust ranking approach is employed for the optimal path selection. Firstly, the paths are ranked in these six features separately. Secondly, a total ranking *S* is given by

$$S=S_{QR}+S_{RS}+S_{Am}+S_{QRm}+S_{RSm}+S_{RR}$$ (6)

where *S*_*X*_ is the ranking of the corresponding feature. The total ranking S fuses the performance of the paths on all features. Thirdly, the optimal path of the tree can be selected by

I=min(S) (7)

where *I* is the index of the optimal path. In the optimal path with *β* FQRS candidates (*β*-layer tree), the first λ (0 < λ ≤ *β*) FQRS candidates can be saved as detected fetal R peaks. Then the same procedure in stage 2 is employed to the subsequent signals (after the last detected fetal R peak). As shown in Algorithm 1, the locations of the FQRS are obtained in each iteration, until the entire AECG is detected.

**Algorithm 1 Procedure of prefix tree-based method**.

**Require**:

1: *G* ← <input matrix> // Input a channel of AECG;

2: *f*_*L*_ ←= 110 // The lower limit of FHR;

3: *f*_*H*_ ←= 180 // The upper limit of FHR;

4: *β* ←= 6 // The number of layers;

5: λ ←= 2 // Number of detected FQRS to be saved;

6: *U* ←= *null* // Vector for detected FQRS;

**Ensure**:

7: **while** NOT reaching the end of *G* **do**

8:  **for** k = 1 to *β* // Tree construction

9:   **if** k ⩵ 1 **then**

10:    *T*_*k*_ ←< *Getlayer*1(*f*_*H*_)>; //construct the layer 1

11:   **else**

12:    *T*_*k*_ ←< *Getotherlayers*(*f*_*L*_, *f*_*H*_)>; //construct other layers

13:   **end if**

14:  **end for**

15:  *F* ←< *Getfeature*(*T*)>; //Feature extraction

16:  *S* ←< *Getranking*(*F*)>; //Optimal path selection

17:  *Q* ←< *GetFQRS*(*S*, λ)>; //Detected FQRS

18:  *U* ← [*U*, *Q*]; //Update the results

19: **end while**

20: **return** *U*

### Stage 3: FP and FN correction

In this work, the construction of the tree can ensure that the range of RR distances (see Eq (8)) is always satisfied within a tree. However, the length of the AECG channel is longer than the tree. We need to construct multiple trees to cover the entire AECG channel. In such a situation, the RR distances between the trees cannot ensure that the range of RR distances is always satisfied. Therefore, after the stage of FQRS detection, a procedure based on the RR distances is implemented for false positive (FP) and false negative (FN) correction. The FP corresponds to the wrongly detected FQRS, and the FN corresponds to the missed detected FQRS.

Specifically, the detected fetal R peaks are evaluated on the RR distances as:

$$d_{L}<RR_{i}<d_{H}$$ (8)

**Fig 6:**
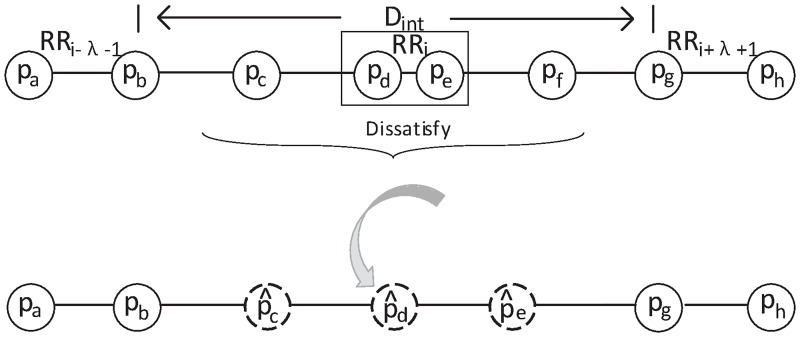
Illustration of correction. After removing 2λ (e.g., λ = 2) detected fetal R peaks, several replicas of the previous detected FQRS would be added in the middle of the considered interval.

Then the detected FQRS which satisfies the Eq (8) could be used to calculate the FHR directly. However, when the Eq (8) is not satisfied, it means that wrong detection occurs within two consecutive trees. Then the detected FQRS of two consecutive trees would be removed (see Fig 6). And the considered interval would be inserted with *m* replicas of the previous detected FQRS in an equal-interval manner. The number of replicas *m* is defined by

$$m=\lfloor \frac{2*D_{int}}{RR_{i-\lambda -1}+RR_{i+\lambda +1}}\rfloor$$ (9)

where *D*_*int*_ is the length of the considered interval, ⌊⋅⌋ is the round down operation.

### Parameter settings

In this paper, a new method based on the prefix tree structure is introduced to FHR measurement. The parameters are described as follows:

- *f*_*L*_ and *f*_*H*_: *f*_*L*_ and *f*_*H*_ indicate the range of FHR. The FHR range should cover the FHR of interest. As indicated in [38], the normal range of FHR is 120 to 160 *bpm*. In order to cover the normal range of FHR, the *f*_*L*_ and *f*_*H*_ are set at 110 and 180, respectively.
- *β* and λ: *β* is the number of layers in the tree. After the optimal path is selected, only the first λ FQRS candidates would be saved as detected FQRS. A deeper tree needs more computational resources to build. In consideration of the limited computational resources, *β* is set at 6. And we have not found significant performance improvement when using deeper trees.
  For the parameter λ, large value is not recommended. When the fetal R peaks are wrongly detected, it is likely that all of the detected FQRS in the selected path are wrongly detected FQRS. In this situation, setting λ with a large value (e.g., six) would increase the number of wrong detections. In this work, after optimizing by grid search within the range of 1 to 6, λ is set at 2. We build a six-layer tree in 0.09 s on Matlab 2017 using a PC with 8 GB RAM and an Intel 2.20 GHz CPU.

### NI-FECG databases

The real AECG records from two public databases are collected to illustrate the efficiency of the proposed method. These databases include the abdominal and direct fetal electrocardiogram database (ADFECGDB) [39] and the Set A of 2013 PhysioNet/Computing in cardiology challenge database (PCDB) [40]. Both databases are available on PhysioNet (https://physionet.org/physiobank/database/adfecgdb/, https://physionet.org/physiobank/database/challenge/2013/). The details of the databases are summarized as follows:

- The abdominal and direct fetal electrocardiogram database (ADFECGDB) contains five records collected from five pregnant women. Each record has 4 abdominal channels and one scalp ECG. The signal lasts for 5 min and is sampled at *f*_*s*_ = 1 kHz. The reference FQRS annotation derived from the scalp ECG is available [39].
- Set A of 2013 PhysioNet/Computing in cardiology challenge database (PCDB) consists of 75 one-minute abdominal records. Data is sampled at *f*_*s*_ = 1 kHz. Each record contains four abdominal channels and the FQRS reference is available. To date, this database is the largest publicly available dataset [40].

As indicated in [19], seven AECG records (a33, a38, a47, a52, a54, a71 and a74) are discarded from the PCDB because of inaccurate reference annotations. In addition, the r04 Ab-1, r07 Ab-1 and r10 Ab-3 are excluded from the ADFECGDB because of severe noise. All records are resampled with fs = 250 Hz.

### Evaluation metrics

In the previous methods (e.g., the TS methods), the FQRS is detected on the residual signals after the MECG is removed. The comparisons between the detected beats and the reference beats are usually used to assess the performances of FQRS detection. When the detected FQRS is within 50 ms of the reference annotation, then the detected FQRS could be considered as a true positive (correctly detected fetal QRS) [9]. Specifically, the sensitivity (*SE*), positive predictive value (*PPV*) and *F*_1_ measure (*F*_1_) are the evaluation metrics typically used to assess the error of FQRS detection [1]. The definitions of the three metrics are given by

$$SE=\frac{TP}{TP+FN}$$ (10)

$$PPV=\frac{TP}{TP+FP}$$ (11)

$$F_{1}=2\times \frac{SE\times PPV}{SE+PPV}=\frac{2\times TP}{2\times TP+FN+FP}$$ (12)

where TP corresponds to the number of true positives. As mentioned earlier, the FP and FN correspond to the number of false positive (falsely detected non-FQRS peaks) and false negative (missed FQRS detections), respectively.

## Results

**Fig 7:**
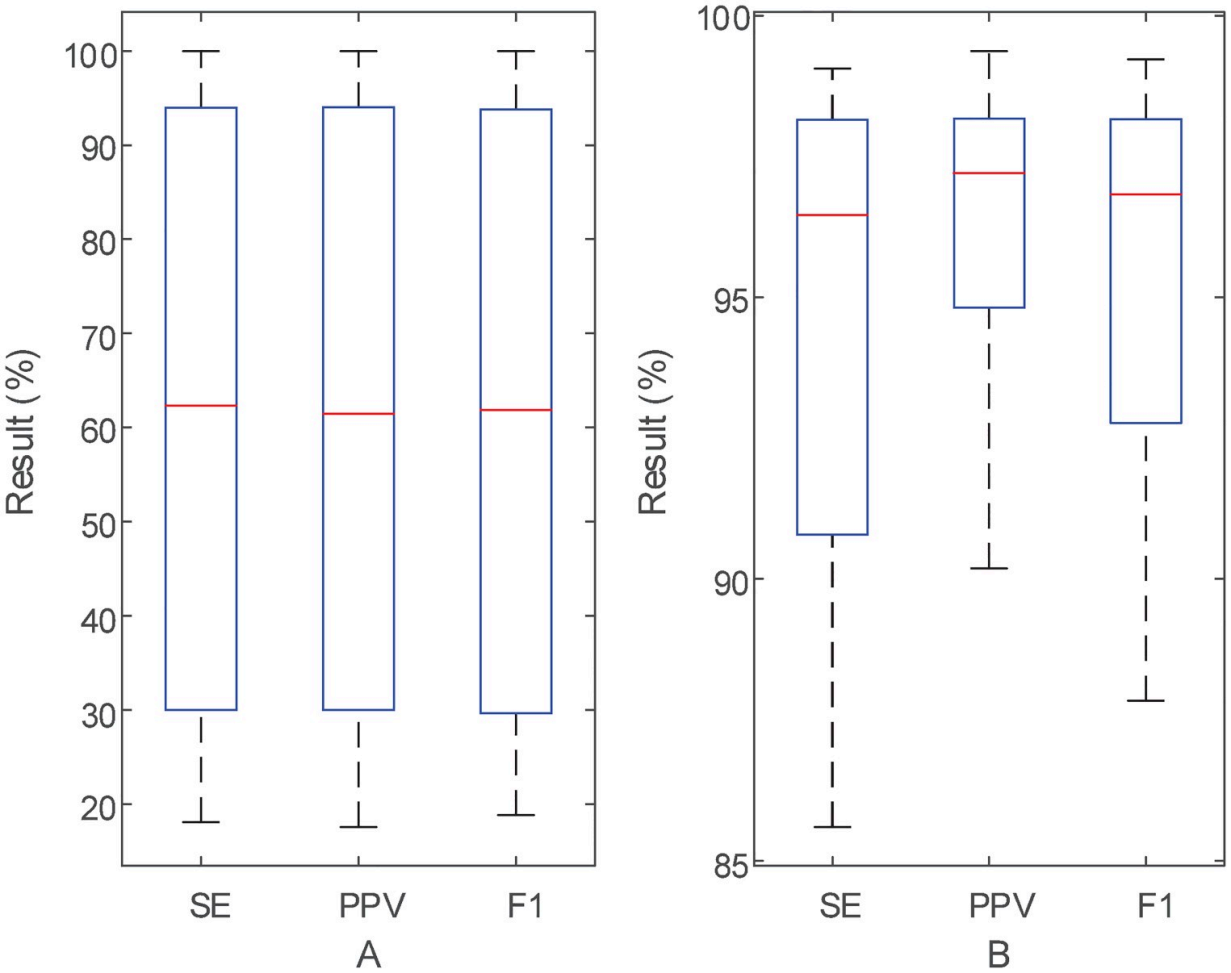
Summary of the results on two databases: A PCDB (Tables 1 and 2), B ADFECGDB (Table 3).

In this study, the real AECG records from the PCDB and ADFECGDB are collected to evaluate the efficiency of the proposed method. Results of the proposed method on PCDB are shown in Tables 1 and 2. Results of the proposed method on ADFECGDB are shown in Table 3. In order to obtain the overall distribution of the metrics, the results on these two databases are visually summarized in Fig 7. It is noted that the results of the proposed method on PCDB are 61.52 ± 30.34 *SE*(%), 61.66 ± 30.06 *PPV* (%) and 61.55 ± 30.20 *F*_1_ (%). And the results of the proposed method on ADFECGDB are 94.72 ± 4.12 *SE*(%), 96.38 ± 2.45 *PPV* (%) and 95.54 ± 3.28 *F*_1_ (%).

**Table 1:** Evaluation results on the PCDB, part 1.

Recordings	*SE* (%)	*PPV* (%)	*F*_1_ (%)	Recordings	*SE* (%)	*PPV* (%)	*F*_1_ (%)
a01 Ab-1	97.01	97.01	97.01	a02 Ab-1	48.99	51.40	50.17
a01 Ab-2	62.69	62.69	62.69	a02 Ab-2	22.30	28.74	25.11
a01 Ab-3	63.43	61.59	62.50	a02 Ab-3	31.65	35.48	33.46
a01 Ab-4	94.78	94.78	94.78	a02 Ab-4	35.57	42.74	38.83
a03 Ab-1	86.49	88.07	87.27	a04 Ab-1	87.50	86.23	86.86
a03 Ab-2	89.19	86.09	87.61	a04 Ab-2	95.00	92.26	93.61
a03 Ab-3	54.62	52.85	53.72	a04 Ab-3	87.50	83.55	85.48
a03 Ab-4	85.27	91.47	88.26	a04 Ab-4	90.83	88.23	89.51
a05 Ab-1	100	100	100	a06 Ab-1	89.93	93.98	91.91
a05 Ab-2	100	100	100	a06 Ab-2	33.09	38.98	35.80
a05 Ab-3	100	100	100	a06 Ab-3	45.22	50.59	47.75
a05 Ab-4	100	100	100	a06 Ab-4	63.31	66.17	64.71
a07 Ab-1	79.65	78.26	78.95	a08 Ab-1	100	100	100
a07 Ab-2	30.09	26.56	28.22	a08 Ab-2	100	100	100
a07 Ab-3	31.86	29.27	30.51	a08 Ab-3	100	100	100
a07 Ab-4	61.16	60.66	60.91	a08 Ab-4	100	100	100
a09 Ab-1	70.33	71.86	71.09	a10 Ab-1	74.88	83.79	79.08
a09 Ab-2	22.12	17.56	19.58	a10 Ab-2	39.01	45.07	41.82
a09 Ab-3	53.98	50.41	52.14	a10 Ab-3	30.92	37.01	33.69
a09 Ab-4	25.62	24.80	25.20	a10 Ab-4	75.56	84.75	79.89
a11 Ab-1	71.48	73.03	72.24	a12 Ab-1	98.44	98.44	98.44
a11 Ab-2	25.95	27.87	26.88	a12 Ab-2	97.66	97.66	97.66
a11 Ab-3	35.11	35.94	35.52	a12 Ab-3	86.44	85.71	86.08
a11 Ab-4	37.40	35.51	36.43	a12 Ab-4	96.88	96.88	96.88
a13 Ab-1	63.94	61.90	62.90	a14 Ab-1	84.48	84.48	84.48
a13 Ab-2	94.02	93.22	93.62	a14 Ab-2	77.59	75.63	76.60
a13 Ab-3	85.47	82.64	84.03	a14 Ab-3	52.59	48.80	50.62
a13 Ab-4	98.17	98.17	98.17	a14 Ab-4	84.48	84.48	84.48
a15 Ab-1	85.71	85.04	85.38	a16 Ab-1	62.89	60.79	61.83
a15 Ab-2	82.54	80.87	81.70	a16 Ab-2	27.43	26.96	27.19
a15 Ab-3	88.10	88.10	88.10	a16 Ab-3	25.66	24.37	25.00
a15 Ab-4	84.13	81.75	82.92	a16 Ab-4	26.55	27.27	26.91
a17 Ab-1	86.84	87.61	87.22	a18 Ab-1	38.46	44.25	41.15
a17 Ab-2	95.12	95.12	95.12	a18 Ab-2	25.00	30.70	27.56
a17 Ab-3	96.75	96.75	96.75	a18 Ab-3	32.31	38.18	35.00
a17 Ab-4	100	100	100	a18 Ab-4	23.08	26.79	24.79
a19 Ab-1	47.06	47.86	47.46	a20 Ab-1	60.16	58.89	59.52
a19 Ab-2	100	100	100	a20 Ab-2	90.16	87.30	88.71
a19 Ab-3	100	100	100	a20 Ab-3	82.13	83.33	82.73
a19 Ab-4	86.55	85.83	86.19	a20 Ab-4	96.72	95.93	96.33
a21 Ab-1	93.65	94.40	94.02	a22 Ab-1	95.73	95.73	95.73
a21 Ab-2	37.04	39.06	38.02	a22 Ab-2	100	100	100
a21 Ab-3	39.26	40.77	40.00	a22 Ab-3	100	100	100
a21 Ab-4	90.37	91.04	90.71	a22 Ab-4	100	100	100
a23 Ab-1	53.18	54.25	53.71	a24 Ab-1	75.79	72.24	73.98
a23 Ab-2	95.76	95.76	95.76	a24 Ab-2	93.46	93.46	93.46
a23 Ab-3	100	100	100	a24 Ab-3	93.91	93.91	93.91
a23 Ab-4	94.92	94.92	94.92	a24 Ab-4	85.05	81.98	83.49
a25 Ab-1	55.56	53.28	54.39	a26 Ab-1	31.09	30.08	30.58
a25 Ab-2	96.58	95.76	96.17	a26 Ab-2	78.91	77.25	78.07
a25 Ab-3	100	100	100	a26 Ab-3	78.29	77.10	77.69
a25 Ab-4	94.02	93.22	93.62	a26 Ab-4	93.80	94.53	94.16
a27 Ab-1	36.98	36.05	36.51	a28 Ab-1	99.36	98.10	98.73
a27 Ab-2	32.22	30.59	31.38	a28 Ab-2	100	98.73	99.36
a27 Ab-3	39.37	35.87	37.54	a28 Ab-3	100	98.73	99.36
a27 Ab-4	32.22	29.18	30.62	a28 Ab-4	100	96.89	98.42
a29 Ab-1	29.30	30.56	29.92	a30 Ab-1	29.70	30.16	29.92
a29 Ab-2	41.49	40.80	41.14	a30 Ab-2	38.94	43.23	40.97
a29 Ab-3	27.19	28.04	27.61	a30 Ab-3	30.30	25.16	27.49
a29 Ab-4	28.42	28.42	28.42	a30 Ab-4	25.76	28.33	26.98
a31 Ab-1	29.13	27.01	28.03	a32 Ab-1	35.92	40.16	37.92
a31 Ab-2	86.61	85.94	86.27	a32 Ab-2	28.03	31.36	29.60
a31 Ab-3	83.46	82.17	82.81	a32 Ab-3	27.46	27.46	27.46
a31 Ab-4	32.20	34.23	33.19	a32 Ab-4	25.76	28.33	26.98
a34 Ab-1	31.90	31.90	31.90	a35 Ab-1	99.34	97.40	98.36
a34 Ab-2	84.43	81.75	83.06	a35 Ab-2	99.34	97.40	98.36
a34 Ab-3	77.05	74.60	75.81	a35 Ab-3	99.34	97.40	98.36
a34 Ab-4	55.17	52.89	54.01	a35 Ab-4	93.38	92.76	93.07
a36 Ab-1	100	100	100	a37 Ab-1	56.06	56.49	56.27
a36 Ab-2	99.36	100	99.68	a37 Ab-2	39.02	42.48	40.68
a36 Ab-3	99.36	100	99.68	a37 Ab-3	20.33	21.14	20.72
a36 Ab-4	96.15	97.40	96.77	a37 Ab-4	79.55	80.77	80.15
a39 Ab-1	55.56	56.92	56.22	a40 Ab-1	23.19	24.24	23.70
a39 Ab-2	53.39	64.95	58.60	a40 Ab-2	87.60	87.60	87.60
a39 Ab-3	25.42	23.26	24.29	a40 Ab-3	26.81	30.58	28.57
a39 Ab-4	88.98	88.24	88.61	a40 Ab-4	97.10	97.10	97.10

**Table 2:** Evaluation results on the PCDB, part 2. At the end of the table, mean scores and the standard deviations (mean ± std) are reported.

Recordings	*SE* (%)	*PPV* (%)	*F*_1_ (%)	Recordings	*SE* (%)	*PPV* (%)	*F*_1_ (%)
a41 Ab-1	47.24	50.42	48.78	a42 Ab-1	22.14	21.98	22.05
a41 Ab-2	73.23	74.40	73.81	a42 Ab-2	96.45	96.45	96.45
a41 Ab-3	26.77	25.76	26.25	a42 Ab-3	25.95	32.08	28.69
a41 Ab-4	24.58	30.21	27.10	a42 Ab-4	97.87	97.87	97.87
a43 Ab-1	31.39	37.37	34.13	a44 Ab-1	100	100	100
a43 Ab-2	34.01	38.17	35.97	a44 Ab-2	100	100	100
a43 Ab-3	19.73	21.48	20.57	a44 Ab-3	100	100	100
a43 Ab-4	26.28	28.13	27.17	a44 Ab-4	100	100	100
a45 Ab-1	22.31	23.20	22.75	a46 Ab-1	50.88	46.03	48.33
a45 Ab-2	62.31	61.36	61.83	a46 Ab-2	47.15	45.67	46.40
a45 Ab-3	56.15	53.68	54.89	a46 Ab-3	23.58	22.48	23.02
a45 Ab-4	62.31	62.79	62.55	a46 Ab-4	24.39	21.58	22.90
a48 Ab-1	23.73	24.35	24.03	a49 Ab-1	100	100	100
a48 Ab-2	81.36	81.36	81.36	a49 Ab-2	100	100	100
a48 Ab-3	78.23	74.05	76.08	a49 Ab-3	99.22	99.22	99.22
a48 Ab-4	92.37	90.83	91.60	a49 Ab-4	100	100	100
a50 Ab-1	23.77	22.31	23.02	a51 Ab-1	42.97	41.98	42.47
a50 Ab-2	25.76	25.95	25.86	a51 Ab-2	27.34	26.72	27.03
a50 Ab-3	29.55	29.77	29.66	a51 Ab-3	64.06	61.65	62.84
a50 Ab-4	28.03	27.82	27.92	a51 Ab-4	43.75	44.09	43.92
a53 Ab-1	23.78	27.64	25.56	a55 Ab-1	24.39	24.79	24.59
a53 Ab-2	79.72	84.44	82.01	a55 Ab-2	87.97	88.64	88.30
a53 Ab-3	22.56	25.84	24.10	a55 Ab-3	84.21	85.50	84.85
a53 Ab-4	100	100	100	a55 Ab-4	38.35	40.16	39.23
a56 Ab-1	29.37	29.84	29.60	a57 Ab-1	54.35	52.82	53.57
a56 Ab-2	29.91	33.02	31.39	a57 Ab-2	37.68	36.11	36.88
a56 Ab-3	34.19	36.70	35.40	a57 Ab-3	30.43	26.92	28.57
a56 Ab-4	25.40	25.00	25.20	a57 Ab-4	21.01	21.10	21.06
a58 Ab-1	35.16	36.29	35.71	a59 Ab-1	23.78	25.00	24.37
a58 Ab-2	66.41	66.67	66.54	a59 Ab-2	20.28	21.64	20.94
a58 Ab-3	40.34	46.15	43.05	a59 Ab-3	27.97	27.78	27.87
a58 Ab-4	28.13	29.27	28.69	a59 Ab-4	22.56	24.79	23.62
a60 Ab-1	28.78	27.21	27.97	a61 Ab-1	26.23	26.89	26.56
a60 Ab-2	26.62	25.52	26.06	a61 Ab-2	97.71	96.24	96.97
a60 Ab-3	22.14	22.14	22.14	a61 Ab-3	49.62	47.79	48.69
a60 Ab-4	25.95	26.15	26.05	a61 Ab-4	96.18	94.74	95.45
a62 Ab-1	25.93	27.34	26.62	a63 Ab-1	26.67	25.81	26.23
a62 Ab-2	100	100	100	a63 Ab-2	33.33	33.59	33.46
a62 Ab-3	100	100	100	a63 Ab-3	30.00	30.25	30.13
a62 Ab-4	94.07	94.07	94.07	a63 Ab-4	34.17	32.28	33.20
a64 Ab-1	28.35	25.35	26.77	a65 Ab-1	24.39	26.32	25.32
a64 Ab-2	31.50	29.41	30.42	a65 Ab-2	78.95	77.21	78.07
a64 Ab-3	26.77	22.08	24.20	a65 Ab-3	18.05	19.67	18.82
a64 Ab-4	27.56	23.49	25.36	a65 Ab-4	80.45	80.45	80.45
a66 Ab-1	26.45	24.81	25.60	a67 Ab-1	68.18	65.22	66.67
a66 Ab-2	95.87	95.08	95.47	a67 Ab-2	67.83	67.83	67.83
a66 Ab-3	98.35	98.35	98.35	a67 Ab-3	85.31	82.43	83.85
a66 Ab-4	97.52	98.33	97.93	a67 Ab-4	82.52	83.10	82.81
a68 Ab-1	28.33	28.10	28.22	a69 Ab-1	87.77	89.71	88.73
a68 Ab-2	27.50	23.40	25.29	a69 Ab-2	99.28	99.28	99.28
a68 Ab-3	26.67	26.02	26.34	a69 Ab-3	90.65	91.30	90.97
a68 Ab-4	31.67	31.15	31.40	a69 Ab-4	92.81	94.16	93.48
a70 Ab-1	66.67	69.57	68.09	a72 Ab-1	98.71	96.75	97.72
a70 Ab-2	90.83	90.08	90.46	a72 Ab-2	99.35	100	99.68
a70 Ab-3	45.83	46.61	46.22	a72 Ab-3	99.35	100	99.68
a70 Ab-4	34.17	32.54	33.33	a72 Ab-4	96.13	97.39	96.75
a73 Ab-1	24.59	26.55	25.53	a75 Ab-1	32.00	27.59	29.63
a73 Ab-2	31.97	33.62	32.77	a75 Ab-2	25.86	21.58	23.53
a73 Ab-3	25.41	24.80	25.10	a75 Ab-3	37.60	33.10	35.21
a73 Ab-4	33.08	34.13	33.59	a75 Ab-4	32.00	29.85	30.89
	*SE*(%)		*PPV*(%)		*F*_1_ (%)		
Overall	61.52 ± 30.34		61.66 ± 30.06		61.55 ± 30.20		

**Table 3:** Evaluation results on the ADFECGDB. At the end of the table, mean scores and the standard deviations (mean ± std) are reported.

Recordings	*SE*(%)	*PPV*(%)	*F*_1_ (%)	Recordings	*SE*(%)	*PPV*(%)	*F*_1_ (%)
r01 Ab-1	98.12	98.27	98.20	r04 Ab-1	-	-	-
r01 Ab-2	98.43	98.59	98.51	r04 Ab-2	91.84	94.45	93.13
r01 Ab-3	99.06	99.37	99.22	r04 Ab-3	90.89	94.49	92.66
r01 Ab-4	98.90	99.22	99.06	r04 Ab-4	89.18	92.89	90.99
r07 Ab-1	-	-	-	r08 Ab-1	97.52	97.83	97.67
r07 Ab-2	95.68	96.30	95.99	r08 Ab-2	97.23	98.14	97.68
r07 Ab-3	95.98	96.14	96.06	r08 Ab-3	96.46	97.20	96.83
r07 Ab-4	98.23	98.07	98.15	r08 Ab-4	96.46	97.20	96.83
r10 Ab-1	90.49	95.25	92.81	r10 Ab-3	-	-	-
r10 Ab-2	90.18	94.93	92.49	r10 Ab-4	85.60	90.19	87.84
	*SE*(%)		*PPV*(%)		*F*_1_ (%)		
Overall	94.72 ± 4.12		96.38 ±2.45		95.54 ± 3.28		

**Fig 8:**
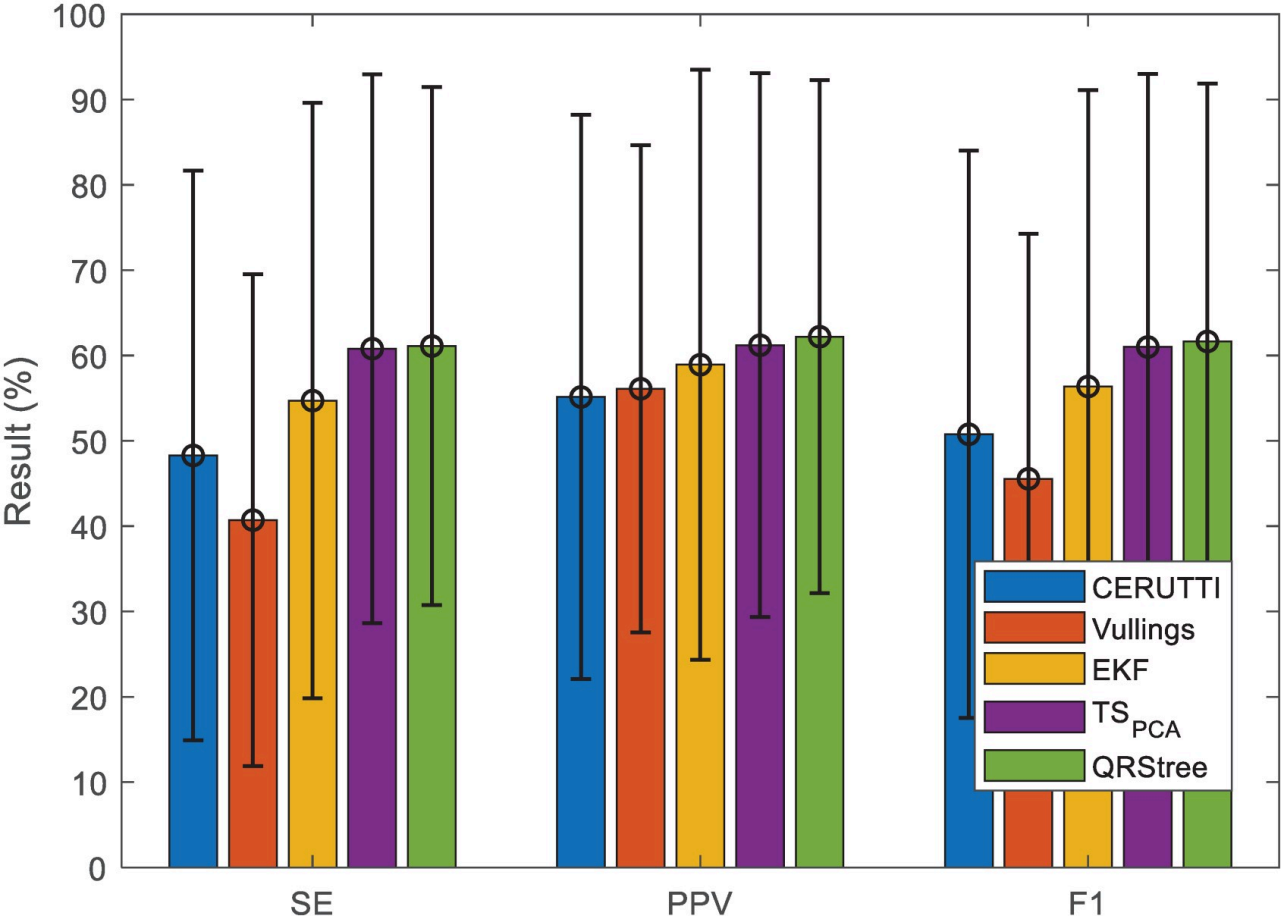
Comparison between the QRStree and four TS methods on PCDB. The results from the Tables 1 and 2 are used. The bars correspond to the mean scores of the corresponding metrics.

**Fig 9:**
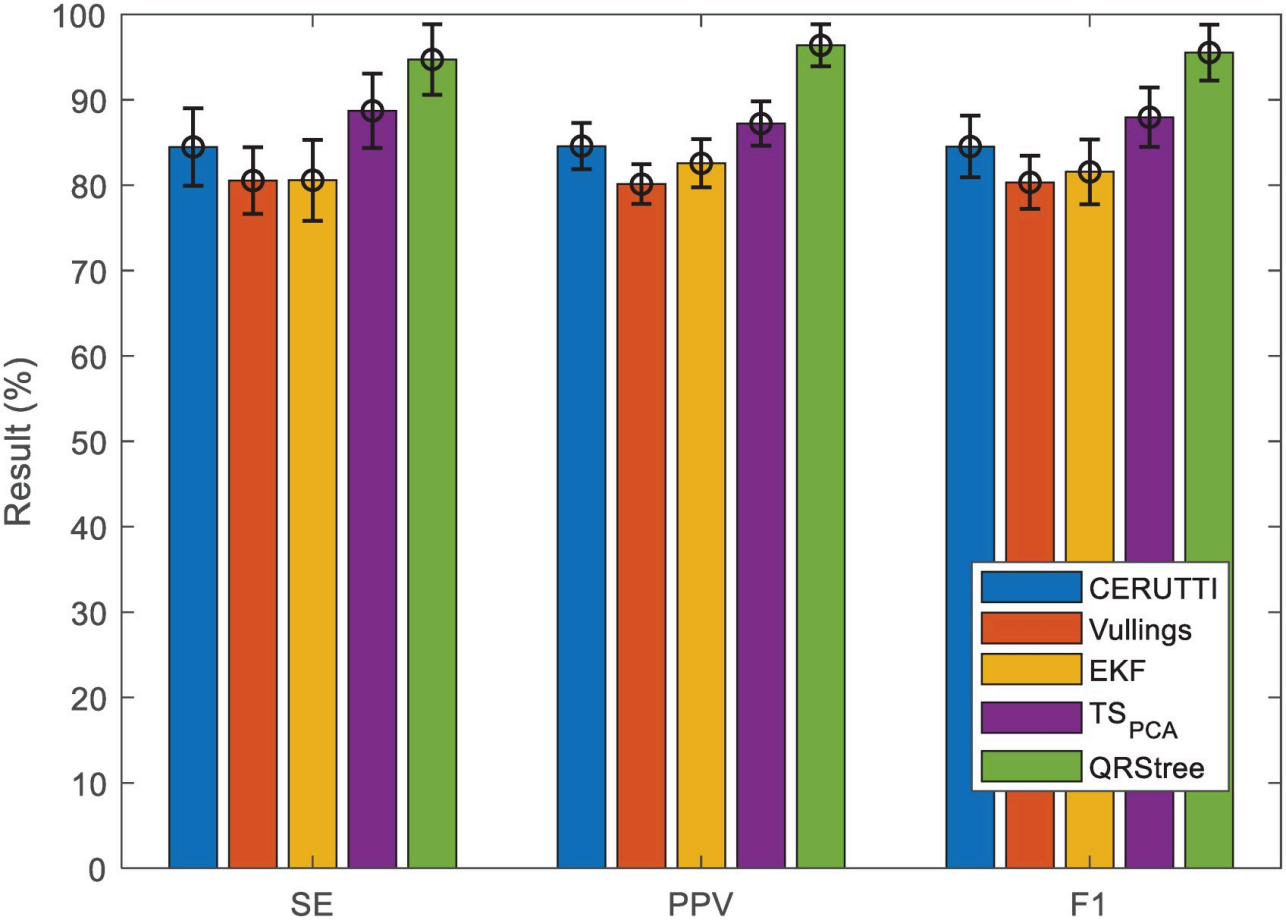
Comparison between the QRStree and four TS methods on ADFECGDB. The results from the Table 3 are used. The bars correspond to the mean scores of the corresponding metrics.

Results of the proposed method are compared with four TS methods [25–27, 37] in Figs 8 and 9. These methods include the Cerutti method (Cerutti), the Vullings method (Vullings), the *TS*_*PCA*_ and the EKF [25–27, 37]. The work of [9] has shown that the TS methods are able to extract the location of FQRS. Within the TS category, the *TS*_*PCA*_ performs the best [9]. These compared methods are implemented on the FECGSYN toolbox [9]. As shown in Figs 8 and 9, among four compared methods, the *TS*_*PCA*_ outperforms other methods. and the result of QRStree is comparable with the state-of-art result reported in the field.

**Fig 10:**
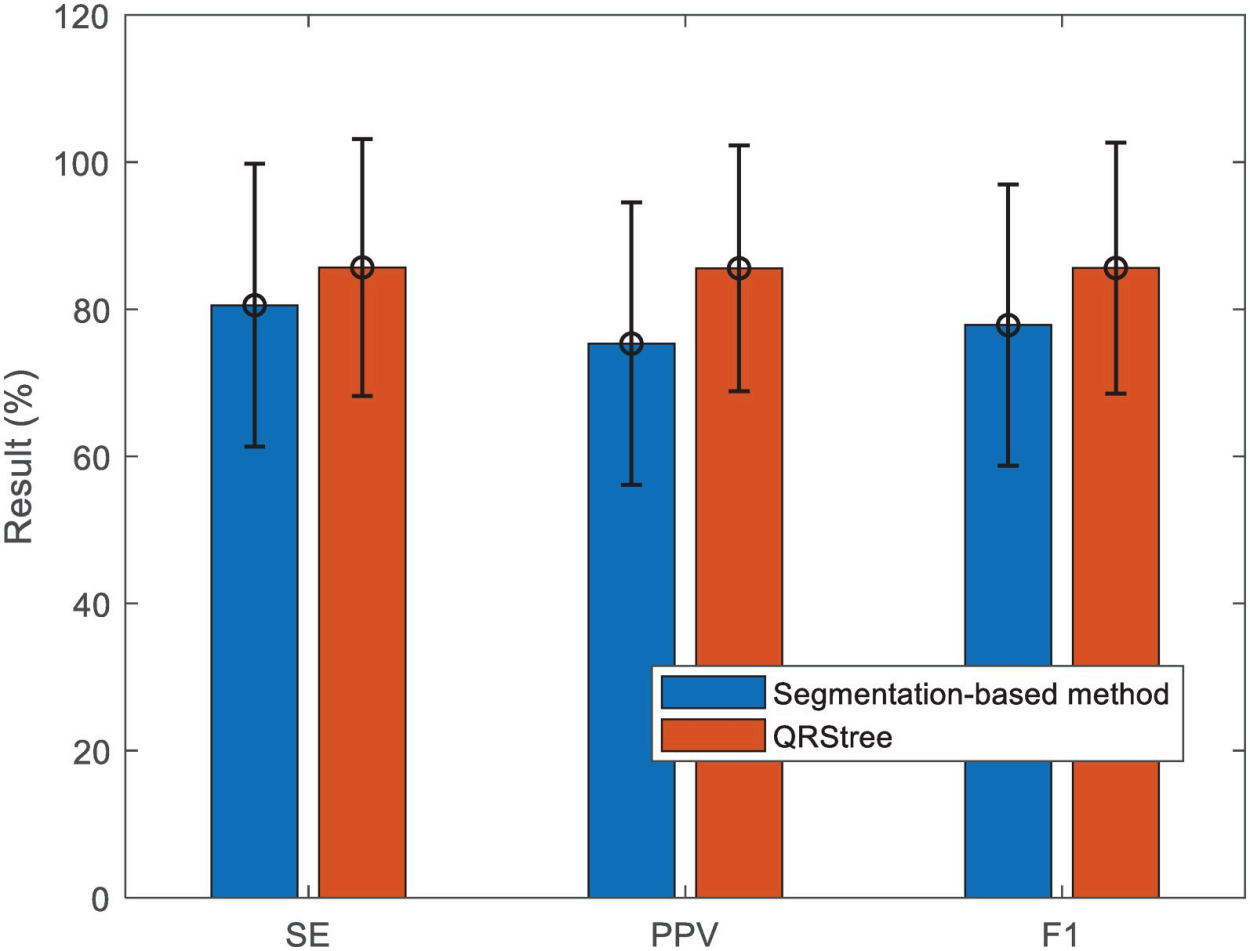
Comparison between the QRStree and the segmentation-based method on seven records of PCDB. The bars correspond to the mean scores of the corresponding metrics.

In this work, we also compare the proposed method with the segmentation-based method [30] (see Fig 10). As indicated in [30], seven records (a01, a02, a03, a04, a05, a06 and a07) from the PCDB are used as the test data, and the result of the best channel is used as the result of the corresponding record. Results show that the QRStree achieves better performance with 85.68 ± 17.48 *SE* (%), 85.57 ± 16.71 *PPV* (%) and 85.61 ± 17.05 *F*_1_ (%).

## Discussions

In this study, a new prefix tree-based method is proposed for FHR measurement from single-channel AECG recording. Tree structure is a good method used to describe the FQRS candidate which will be stored in one node in the tree. In the field of NI-FECG signal processing, the frequency and temporal overlap of the MECG and FECG signals makes the FHR measurement challenging. However, since the tree structure is built on the range of FHR, only the local maxima satisfied the range will be collected for use, that is, it can skip the maternal R peaks which are not satisfied the range of FHR. As a result, the FQRS and MQRS become ‘heart rate separable’. It means that the proposed method will not be affected by the large amplitude of MECG, which is the predominant interference source.

The prefix tree in this work is a complete prefix tree, which includes all the possible combination of the FQRS candidates. It is a highly compact tree structure that enables efficient mining of the inter-QRS correlation between the FQRS. Specifically, all the real fetal R peaks are laid in the tree structure, in which the path consisted of real fetal R peaks can be selected. Such that, it has potential to reduce the occurrence of miss detections.

In addition, the proposed method only requires single-channel AECG. Compared with the algorithms which require multiple channels, it is a considerable advantage from the standpoint of pregnant women. It is worth noting that focusing on one-lead FQRS detection techniques facilitates the production of low-cost and easy-to-use devices for intrapartum and antepartum monitoring.

Results show that the proposed method achieves a good performance. Key to the good performance is that the proposed approach can effectively extract the inter-QRS correlation between the FQRS. This work shows an interesting way for FHR measurement without degrading the FECG signal. The location of FQRS is directly extracted from the AECG signal, such that the proposed approach has the potential to provide information to extract the original waveform of FQRS from the AECG.

**Fig 11:**
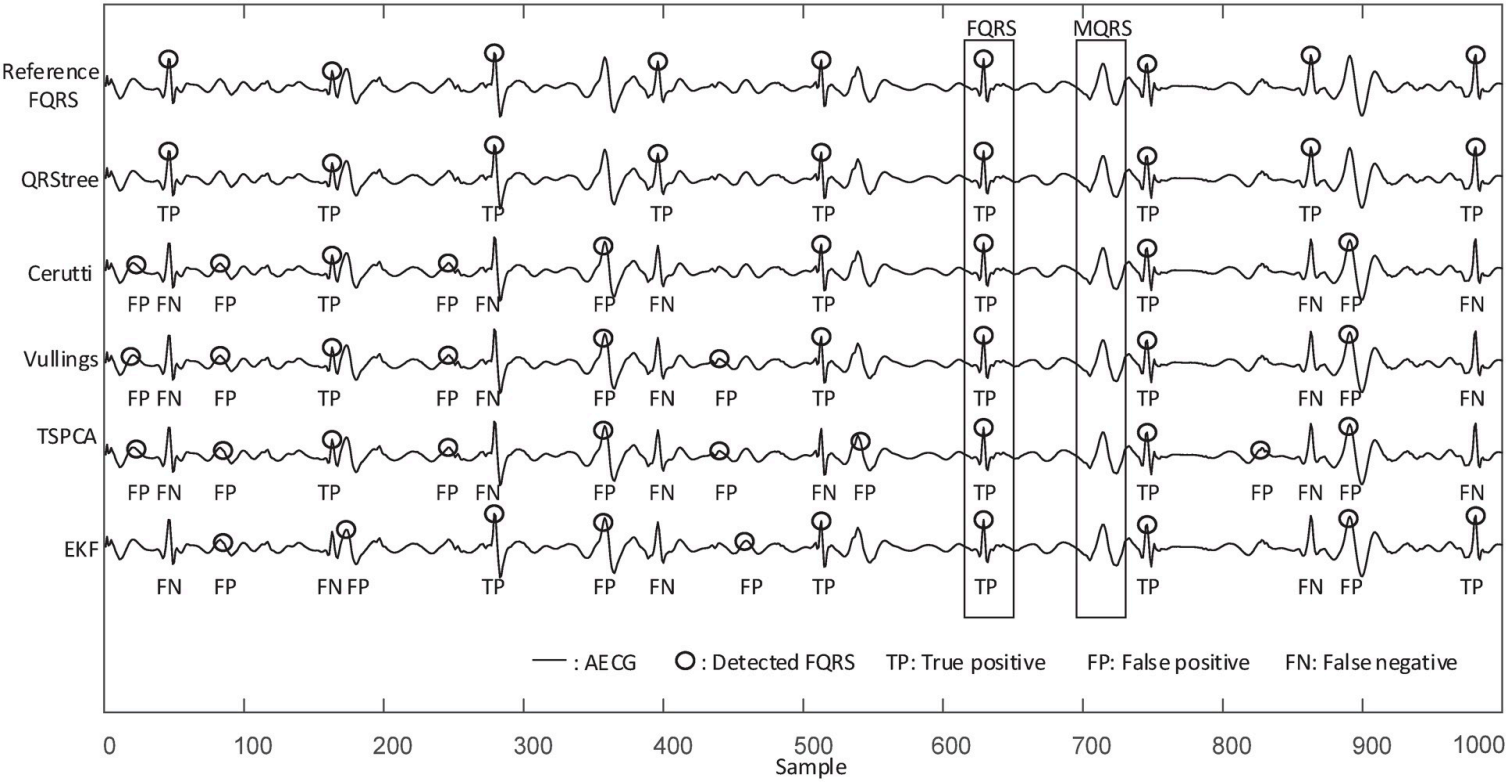
Examples of results with different methods when the FECG and MECG amplitudes are comparable. The performance of TS methods in FQRS detection is reduced (see the FP and FN). However, the QRStree still detects the FQRS effectively. Data is collected from the r01 Ab-2 of ADFECGDB.

By cancelling the MECG, numerous techniques make FHR extraction possible. However, the TS methods require the accurate location of the MQRS for MECG elimination. Otherwise, the MQRS waveform can not be sliced and aligned. It should be noted that obtaining the location of the MQRS is not always easy. As shown in Fig 11, it is difficult to access the location of the MQRS when the FECG and MECG amplitudes are comparable. Consequently, the performance in MECG elimination is reduced, and a reliable FHR measurement can not be obtained. In the proposed QRStree, there is no need for such prior information. Such that the FHR measurement can be achieved directly from the abdominal channel without eliminating the MECG waveform, so the proposed QRStree avoids the problems related to MECG elimination such as MQRS slicing and alignment.

**Fig 12:**
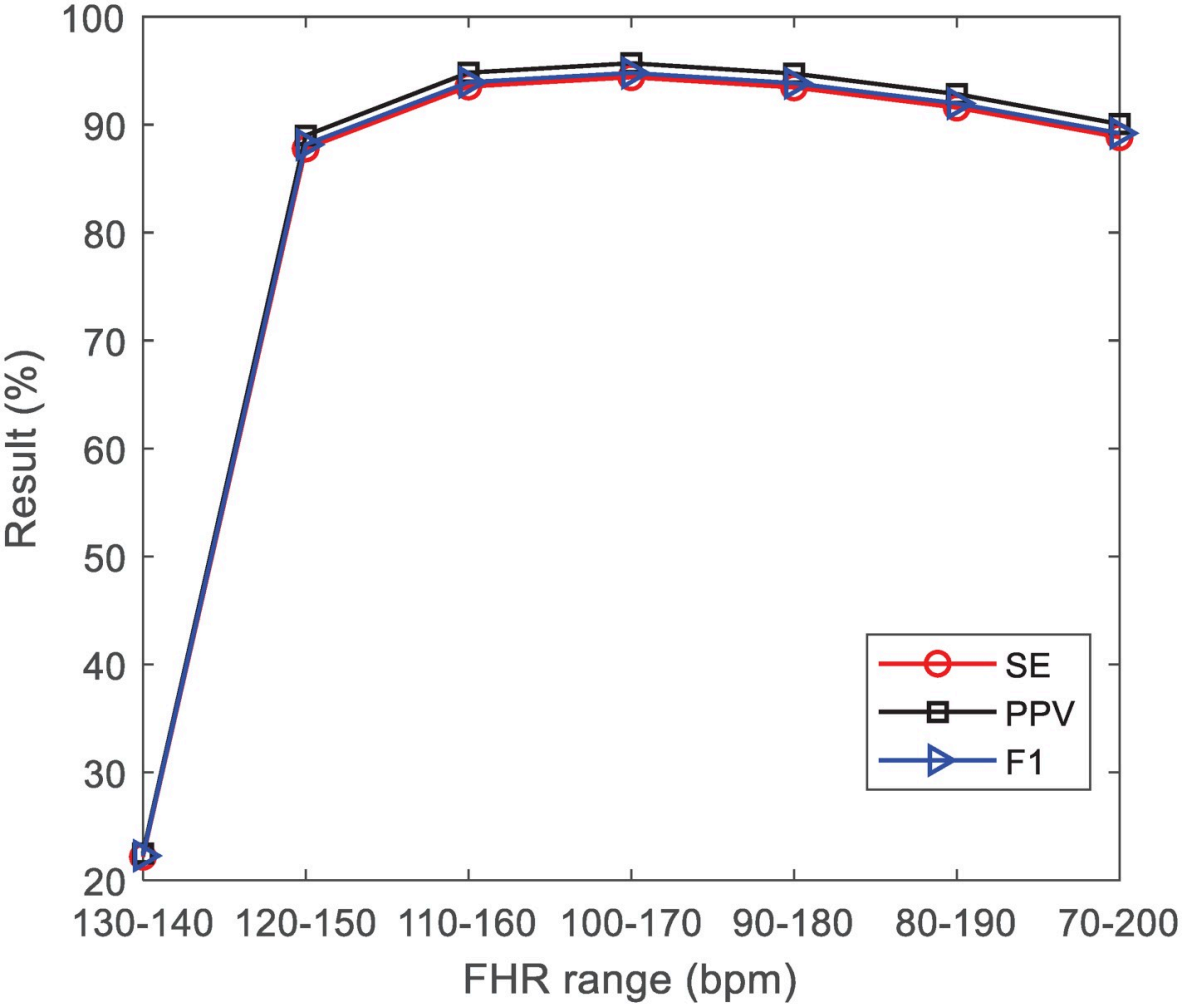
Mean scores of SE, PPV and *F*1 with different FHR ranges. Data is collected from the ADFECGDB. In order to obtain a satisfied result on FHR measurement, the FHR range should cover the FHR of interest.

In order to evaluate the influence of FHR range (*f*_*L*_ and *f*_*H*_) on FQRS detection, we implement a series of contrast experiments with different parameter sets (see Fig 12). Firstly, the FHR range is set at 130-140 *bpm*. Then we broaden the FHR range in steps of 20 *bpm*. It is noted that the FQRS can not be detected by narrow FHR range (e.g., 130-140 *bpm*), since the FHR range has not covered the FHR of interest. As the FHR range becomes broader, the performance has been improved accordingly (e.g., 100-170 *bpm*). However, after the FHR of interest is covered, the performance degrades slowly as the FHR range broadens, since the number of paths is increased and it would raise the risk of misdetections (e.g., 70-200 *bpm*).

In this work, the main objective is to provide a novel method for FQRS detection directly from the AECG without canceling the MECG. And the results show that the proposed method is effective in the task of FQRS detection. However, one limitation of the propose method is that the performance is reduced when the AECG signal is affected by severe noise. In this situation, the methods based on MECG elimination (e.g., the TS methods) can obtain better performance. It is expected that some noise components are removed in the process of MECG elimination. In the future, it is worth to investigate the potential of the proposed method on different noise levels by using synthetic data.

## Conclusions

In this study, a novel framework is presented to FQRS detection from single-channel AECG. Unlike the previous studies based on the elimination or separation of MECG, the proposed QRStree detects the location of FQRS directly from the AECG. Specifically, the FQRS is connected by the range of FHR. And these connections are organized and stored in a simple, but yet powerful tree structure. The results show that the proposed method exhibits a good performance on the task of FQRS detection. This work provides a new perspective for the development of FHR measurement.
